# Exploration of the center of mass kinematics correlation with established gait measures in post-spinal cord injury

**DOI:** 10.3389/fresc.2026.1745887

**Published:** 2026-05-11

**Authors:** Gabrielle C. Labrozzi, Madelaine K. Blincoe, Lisa M. Lombardo, Nathaniel S. Makowski, Musa L. Audu, Ronald J. Triolo

**Affiliations:** 1 Department of Biomedical Engineering, Case Western Reserve University, Cleveland, OH, United States; 2 Motion Study Laboratory, Louis Stokes Cleveland Department of Veterans Affairs Medical Center, Cleveland, OH, United States; 3 Department of Physical Medicine & Rehabilitation, MetroHealth System and Case Western Reserve University School of Medicine, Cleveland, OH, United States

**Keywords:** center of mass, clinical outcome measures, gait assessment, spinal cord injury, walking performance

## Abstract

**Introduction:**

Walking mobility is important for health and quality-of-life after spinal cord injury (SCI). Outcome metrics monitoring rehabilitation progress typically focus on gait speed, assistive technologies, and muscle strength, but do not address all gait determinants in a single metric which limits understanding of gait deviations and compensatory strategies. Center of mass (CoM) is a global parameter reflecting whole-body movement and motor control. We hypothesized CoM kinematics might quantitatively differentiate between clinically accepted ambulatory categories and correlate with clinical gait performance metrics.

**Methods:**

We collected CoM and six established clinical gait assessments from five ambulatory individuals with incomplete SCI over four sessions. Single-sine coefficients of a Fourier Series model were optimized via a non-linear gradient-based optimizer to compute CoM symmetry indices in the mediolateral, anteroposterior, and inferiosuperior directions. We computed correlations between the indices and clinical assessments with a Spearman Correlation Analysis (***α*** = 0.05).

**Results:**

CoM differentiated across impairment levels and uniquely captured deviations from neurotypical gait. Symmetry indices strongly or moderately correlated with 10-Meter Walking Test, 2-min Walking Test, Lower Extremity Motor Score and SCI-Functional Ambulation Inventory depending on the CoM direction (|ρ|>0.5). Two clinical measures were weakly correlated across all directions: Walking Index for Spinal Cord Injury II and Mini-Balance Evaluation Systems Test. Mediolateral and anteroposterior CoM correlated with more measures (three) than the inferiosuperior direction (one).

**Conclusions:**

CoM appears to reflect the multidimensionality of gait and clarify some of the deficiencies of clinically accepted measures. These findings suggest CoM analysis is clinically relevant, and supports further exploration of symmetry indices as figures of merit for gait performance and informing rehabilitation interventions post-SCI.

## 1 Introduction

Walking has consistently been reported as a top priority for improvement by people with spinal cord injury (SCI) (1–3) and impacts independence and greatly benefits overall health (4). Neurotypical (NT) gait involves the entire body with upper and lower extremities contributing to forward propulsion and maintaining postural stability (5). Balance, strength, coordination, and sensation are often compromised by SCI (6), which can negatively impact gait stability, quality and speed (7). Various compensatory strategies and assistive technologies (AT) can partially ameliorate these deficits, which require rigorous and careful measurement to document their effects (6).

Established clinical assessments (Table 1) for various aspects of walking impairment can be categorized into continuous and categorical measures (8). The 10-Meter Walking Test (10MWT) and 6-Minute Walking Test (6MWT) are continuous measures that record the speed or distance travelled within a certain period. While valid metrics (8), they do not address underlying compensatory strategies (9) and are not specific to the causes of deviations from NT gait (i.e., vastly different walking mechanics can yield similar speeds). The Functional Ambulation Category (FAC), Walking Index for SCI II (WISCI-II), Mini-Balance Evaluation Systems Test (Mini-BESTest), and Lower Extremity Motor Score (LEMS) are categorical measurements that score overall walking ability, AT usage (i.e., braces, forearm crutches, etc.), balance, and muscle strength (8). The FAC classifies walkers into broad categories: non-ambulator, physiological, household, and community ambulators based on self-report and observation (11) or gait speed (12). The SCI-Functional Ambulation Index (SCI-FAI) is both a continuous and categorical measure that identifies gait deviations by rating spatiotemporal parameters (8) without accounting for compensatory strategies. The Mini-BESTest measures balance and accounts for AT use by penalizing the score (10). While all these metrics are valid and reliable in their individual contexts, multiple assessments are required to understand and quantify all gait determinants and comprehensively evaluate/screen a patients gait performance and document rehabilitation progress. These assessments individually measure aspects of overall progress, but do not detect gait deviations nor compensatory strategies. Furthermore, improvements in certain assessments may not be reflected in others nor necessarily mean the individual is approaching a NT walking pattern. Regaining normal walking function without assistive devices is a top priority for people with SCI (2, 3). Thus, a global parameter that could be utilized for an initial, overall, scoping analysis and form a foundational baseline is required upon which progressively more detailed and specific analyses could be performed by clinicians. This parameter may capture multiple aspects of gait in a single measure, which might serve to complement and integrate the established individual metrics.

**Table 1:** Summary of established clinical gait assessments.

Outcome measure	Continuous/categorical	Brief description	Pros	Cons
10MWT	Continuous	Records gait speed across 10-meters	Valid and Reliable for SCI (8) Simple Metric	Unknown floor and ceiling effects (9). No information on compensatory strategies.
6MWT	Continuous	Records total distance (meters) in 6 min.	Valid and Reliable for SCI (8) Simple Metric	Unknown floor and ceiling effects (9). No information on compensatory strategies.
SCI-FAI	Continuous/Categorical	Records total distance (meters) in 2 min. A 39-point test that rates walking ability, usage of assistive technology and gait quality	Identifies gait deviations Valid metric for SCI.	Floor and ceiling effects (9). Limited Precision Does not account for compensatory strategies.
FAC	Categorical	Categorizes ambulation into four gross categories.	Categorizes on a global scale	Overlooks gait quality/deviations
WISCI-II (8)	Categorical	A 21-point scale that measures walking dependence on assistive technology.	Valid metric of functional capacity.	Does not explain gait deviations due to injury Floor and ceiling effects (9).
Mini-BESTest (10)	Categorical	A 28-point test that measures static and dynamic balance through 14 tasks.	No ceiling effects. Enables the usage of assistive devices. Valid Metric for SCI	Does not explain gait deviations due to injury.
LEMS (9)	Categorical	A 50-point test that measures strength of muscle movements.	Used for classification of SCI Correlated with lower extremity capabilities	Ceiling Effects Limited Response to muscle changes

10MWT, 10Meter Walking Test; 6MWT, 6Minute Walking Test; SCI-FAI, Spinal Cord Injury Functional Ambulation Index; FAC, Functional Ambulatory Category; WISCI-II, Walking Index for Spinal Cord Injury II; Mini-BESTest, Mini Balance Evaluation System Test; LEMS, Lower Extremity Motor Score.

Center of Mass (CoM) kinematics reflect whole-body movement in space, representing the point about which the body would rotate if concentrated at a single location (13) and can be computed analytically from segmental motion and mass parameters. Kinematics of the CoM in anteroposterior (AP), mediolateral (ML), and inferiosuperior (IS) directions follow well-defined sinusoidal trajectories during NT walking, and significantly deviate from this pattern in pathological gait (14, 15). Thus, CoM can indicate overall gait quality and reflect the directional influence of over- or under-activity and discoordination of various muscle groups (16). Although researchers have examined CoM excursions during walking post-SCI in ML (17) and IS (18) directions independently, all three components are important to gait, with metabolic energy expenditure thought to be prioritized in the IS and AP directions of CoM movement (19), forward propulsion in the AP, and stability and balance in the ML (17).

The CoM profile follows a characteristic “bowtie” shape during slow walking that evolves into a “U” shape with increasing gait speed (13). To quantify these behaviors, Minetti et al. developed a mathematical model consisting of a truncated Fourier Series with six harmonics (20). The model parameterized the CoM so whole-body information regarding various pathologies such as asymmetry, which is prevalent post-SCI, could be extracted (21). While successfully applied to NT treadmill walking, it has yet to be demonstrated and utilized in overground gait post-SCI.

The purpose of this study was to characterize three-dimensional CoM kinematics during post-SCI gait with respect to asymmetry, strength, assistive devices (ADs) and gait speed by adapting the methods developed by Minetti et al. and explore correlations with various established clinical gait assessments. We hypothesized that CoM kinematics can discriminate between at least three ambulation categories with a 0.70 or greater correlation with accepted clinical gait measures. Thus, quantitative CoM analysis may simultaneously reflect multiple determinants of gait and complement accepted clinical assessments that each focus on a single or smaller number of specific measurement domains.

## 2 Methods

### 2.1 Experimental procedure

Five ambulatory individuals with chronic incomplete SCI (iSCI) of varying injury levels and severity, and no vestibular compromise, uncontrolled spasticity, or interfering musculoskeletal issues other than paralysis (Table 2) participated in four sessions of quantitative gait assessment. Ambulation categories were assigned based on the Hoeffer et al. definition (11) reflecting self-reported daily function and later adjusted objectively based on experimental measurement of gait speed (12). This identified one physiological (SCI01), two household (SCI02, SCI03), and two community (SCI04, SCI05) ambulators with varying idiosyncratic limitations. Additionally, one NT individual (NT01) participated in a single walking session. All participants signed consent forms approved by the Institutional Review Board of the Louis Stokes Cleveland VA Medical Center (protocol 1591730, approved 3/11/2021).

**Table 2:** Demographics for neurotypical individual (NT) and individuals with spinal cord injury (SCI).

Subject	Gender	Age (yrs)	Height (cm)	Weight (kg)	Months since Injury	AIS Score	Injury Level	Ambulator Category	Assistive Device(s)
NT 01	M	24	181.6	70.3	–	–	–	–	–
SCI 01	F	63	167.6	52.2	492	C	C4	Physiological	Forearm Crutches
SCI 02	M	47	180.3	114.8	48	D	T11	Household	Forearm Crutches
SCI 03	M	48	190.5	108.9	73	C	C6	Household	Forearm Crutches
SCI 04	M	64	172.7	121.9	10	D	T1	Limited Community	Cane (Right Side)
SCI 05	M	29	182.9	83.9	14	C	L2	Community	Bilateral AFOs, Forearm Crutches

AIS is on a 5-point scale from A-E where A is the absence of motor and sensory function and E is an injury without discernable neurological deficits (22). AIS, American Spinal Injury Association (ASIA) Impairment Scale; AFOs, Ankle Foot Orthoses.

**Figure 1:**
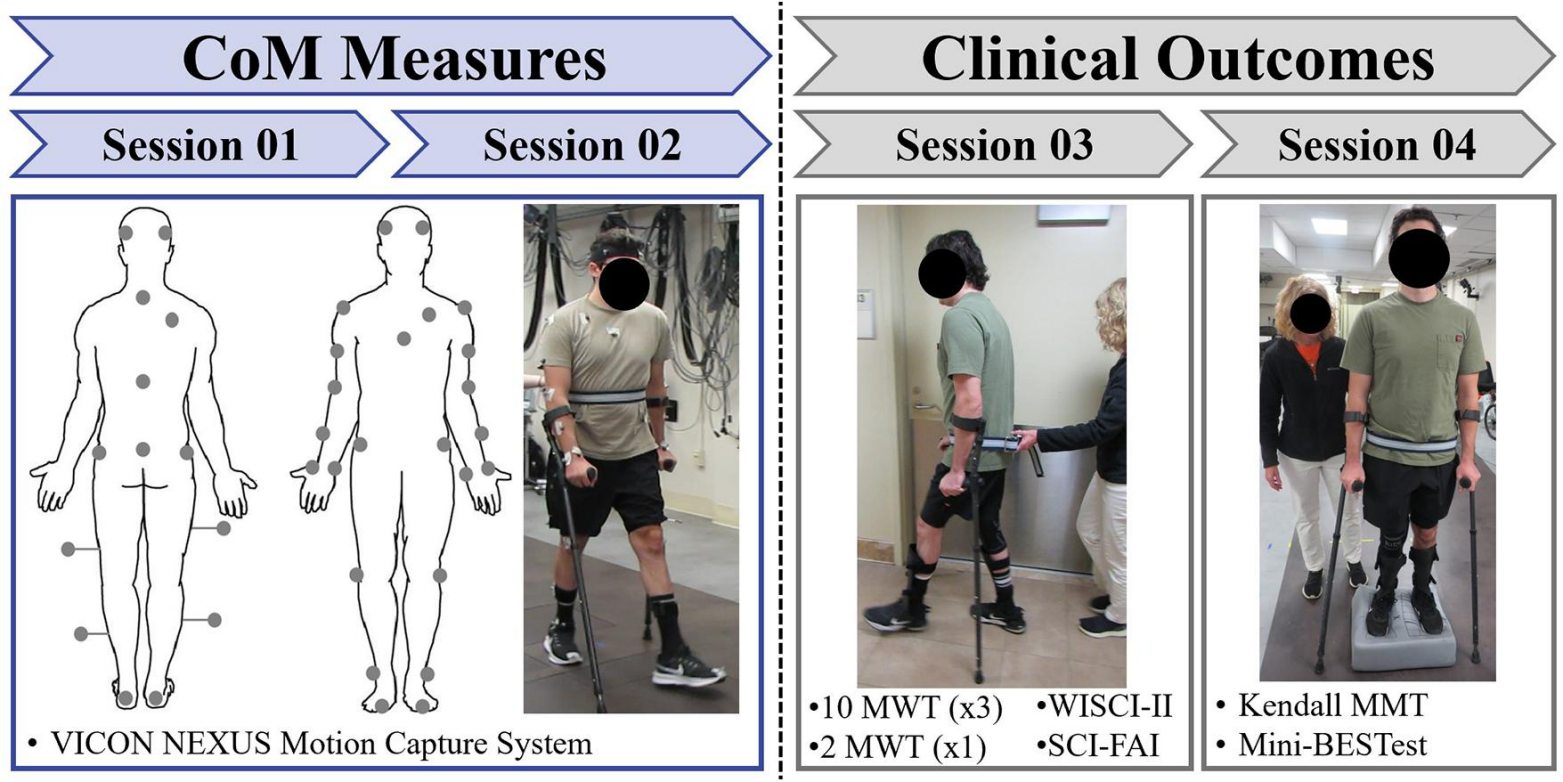
The experiment workflow. The experiment was broken into four sessions. We collected positional data during the first two sessions to compute the center of mass (CoM) positions (LEFT). The gray circles on the human diagram are the reflective marker positions. The next two sessions focused on clinical outcome measures (RIGHT). Reflective markers were not used for these sessions. Session 03 focused on gait speed and quality (the 2MWT shown above). We collected balance and muscle strength information during the fourth session. One of the balance tests from the Mini-BESTest examination is shown above. 10MWT, 10Meter Walking Test; 2MWT, 2Minute Walking Test; WISCI-II, Walking Index for Spinal Cord Injury II; SCI-FAI, Spinal Cord Injury Functional Ambulation Index; MMT, Manual Muscle Testing; Mini-BESTest, Mini Balance Evaluation System Test.

During the first two sessions, subjects completed a total of 40 walking trials along a 10-m walkway at their preferred speeds while wearing a modified Plug-in Gait 38-reflective marker set (Figure 1, **LEFT**) and using their typical ADs (Table 2). Marker positions were captured at 100 Hz via a VICON 16-camera motion capture system (Oxford Metrics, Oxford, UK) from which CoM kinematics were derived using subject-specific segmental mass parameters (23). SCI01 required rest intervals between every trial, and all others were given five-minutes rest after the first 10 trials. To ensure safety, a physical therapist provided contact guarding. The NT individual completed five walking trials at his preferred speed with the same marker and camera setup.

Clinical assessments were collected over the last two sessions (Figure 1, **RIGHT**) to minimize potential confounds from fatigue. The third session included the 10MWT, 6MWT, WISCI-II, and SCI-FAI, again with their typical ADs at their preferred walking speeds. We derived values for the 2-Minute Walking Test (2MWT) (9) from the first two minutes of the 6MWT and applied the measure directly to SCI01 who was unable to walk uninterrupted for six minutes. The fourth session consisted of the Mini-BESTest, and a subset of Kendall Manual Muscle Test (MMT) (24) from which the LEMS was computed (25). One physical therapist scored all outcome measures for each participant to eliminate inter-rater variability.

### 2.2 Center of mass profile

VICON marker positions from a modified Plug-in Gait reflective marker set (23) and anthropometric tables (26) were used to analytically compute the whole-body CoM position. We applied the methods of Labrozzi et al. to filter, and transform CoM position to the body reference frame (27). Data were then separated by stride, defined as the interval between successive right initial contacts, yielding at least 100 strides per participant which is equivalent to the quantity reported by *Minetti et al*. (20). Inspired by the methods reported in Minetti et al.*,* we computed the symmetry indices in the ML $\left(S_{\mathrm{ML}}\right)$, AP $\left(S_{\mathrm{AP}}\right)$, and IS $\left(S_{\mathrm{IS}}\right)$ directions. This procedure is outlined in the supplementary materials (50, 51).

### 2.3 Statistical analysis

To explore the correlation between CoM symmetry and clinical assessments, we performed a Spearman correlation analysis since it is robust to outliers (17) and useful for nonnormally distributed/nonlinear data (28). *S*_ML_, *S*_AP_, *S*_IS_ were compared to the average speed from the 10MWT (m/s), distance from the 2MWT (meters), and the total scores from each of the WISCI-II, SCI-FAI, Mini-BESTest, and LEMS. When interpreting the results, a Spearman correlation coefficient (|ρ|)>0.7 was considered strong, 0.50–0.70 moderate, and <0.5 weak (17). We used an *α* = 0.05 to test for significance where *p* < 0.05 indicates a correlation is significantly different than zero (29).

## 3 Results

### 3.1 Clinical outcome measures

**Figure 2:**
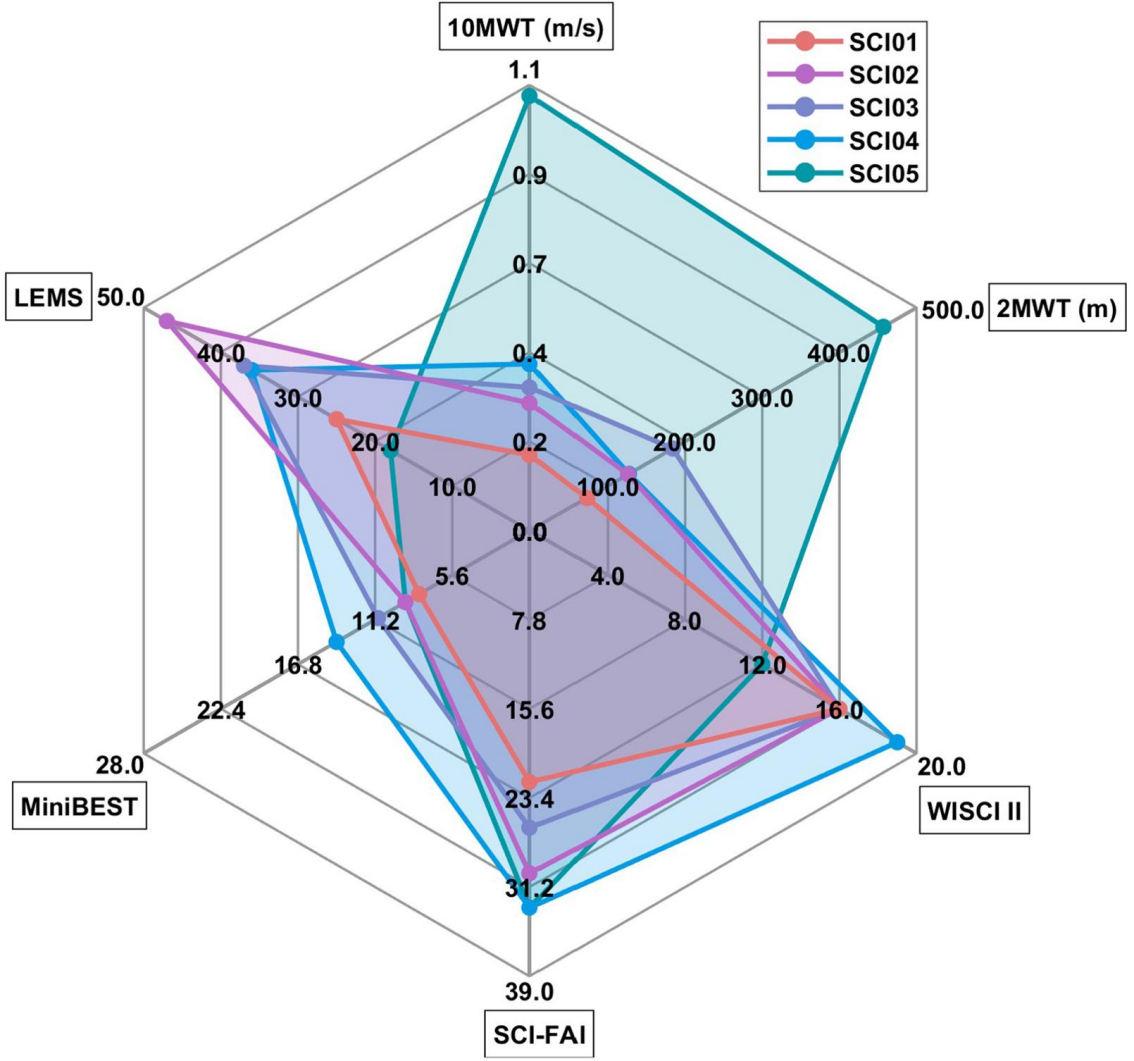
A spider plot of six clinical outcome measures starting from the top and working clockwise: 10-meter walking test, 2-min walking test, walking Index for SCI II, SCI functional ambulation inventory, Mini-balance evaluation systems test. The 10MWT is represented in speed, and the 2MWT in meters. 10MWT, 10Meter Walking Test; 2MWT, 2Minute Walking Test; WISCI-II, Walking Index for Spinal Cord Injury II; SCI-FAI, Spinal Cord Injury Functional Ambulation Index; FAC, Functional Ambulatory Category; Mini-BESTest, Mini Balance Evaluation System Test; LEMS, Lower Extremity Motor Score.

The six clinical assessments for the five participants with iSCI are depicted in Figure 2. Subject performance was not always in order of the assigned FAC and varied across assessments. For instance, SCI05 was the fastest walker (1.07 m/s) with the highest SCI-FAI score (30) but had the lowest LEMS (9) and WISCI-II (10) scores. SCI02 was one of the slowest walkers (0.31 m/s) but exhibited the highest LEMS (31). Areas enclosed in the spider plots (shaded regions) of Figure 2 were computed as a single figure of merit for subject walking performances across all clinical assessments normalized by their maximal allowable values. The computed areas of the shaded regions were 0.43, 0.74, 0.75, 0.95 and 1.16 for SCI01-05 respectively out of a maximum value of 2.60, which coincided with the order of increasing FAC.

### 3.2 Center of mass profiles

**Figure 3:**
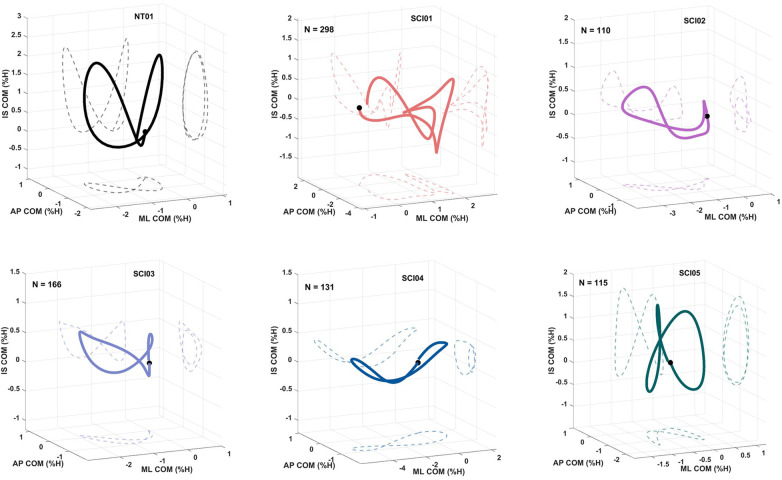
The 3D representations of the center of mass (CoM) profiles for NT01 and SCI01-05. The solid line shows the average CoM profile over all strides (N), and dashed lines are projections onto each anatomical plane. Each profile is normalized and depicted as a percent of subject height (%H). The black dot represents the starting point (right initial contact). Note, the separation between the CoM profiles do not begin and end at the same point due to the intrinsic variability in all biological systems, and natural step-to-step differences. ML, mediolateral; AP, anteroposterior; IS, inferiosuperior.

The normalized CoM profiles for each participant (NT01, SCI01-05) are illustrated in Figure 3. By visual inspection, the CoM profile differentiated across ambulatory categories with the least impaired participant (SCI05) exhibiting the CoM profile most closely resembling NT, and the most impaired (SCI01) showing the greatest deviation. The CoM profile from NT01 and SCI05 (community ambulator) were visually the most symmetrical, followed by SCI03/SCI04, SCI02, (limited community and household ambulators) and SCI01(physiological ambulator) which is the order of increasing impairment. The lateral sway changed observably across participants. NT01 and SCI02-04 shifted towards the stance limb (an initial decrease in the ML CoM), but SCI01 and SCI05 shifted towards the swinging limb.

### 3.3 Center of mass parameterization

The R^2^ values from the cross validation exceeded the criterion (0.70) with three harmonics, or terms, in all CoM directions for most participants (Supplementary Table S1). Only the IS R^2^ for SCI01 did not exceed the criterion until a fourth harmonic was added. Overfitting, as evidenced by decreases in the R^2^ values, began with the fifth harmonic. We therefore chose to use three harmonics when applying the model to all strides and computing the symmetry indices for each subject. Supplementary Figure S1A illustrates the effects of harmonics on the model estimation accuracy for SCI03.

Table 3 contains the Spearman correlation coefficients between the CoM symmetry indices and the clinical assessments. CoM symmetry indices correlated with two-thirds of the clinical measures (10MWT, 2MWT, SCI-FAI, and LEMS), with each CoM direction correlating with a unique combination. For instance, $S_{\mathrm{ML}}$ strongly and positively correlated with speed (10MWT and 2MWT) and moderately with gait quality (SCI-FAI), while $S_{\mathrm{AP}}$ and $S_{\mathrm{IS}}$ both had strong, negative correlations with the muscle strength score (LEMS). $S_{\mathrm{AP}}$ also moderately correlated with gait quality. CoM symmetry did not correlate with utilization of forearm crutches, braces, and/or cane (WISCI-II), nor balance (Mini-BESTest). The correlation between $S_{\mathrm{ML}}$ and 2MWT was the only one found to be significantly different than zero statistically (*p* = 0.017).

**Table 3:** Correlation coefficients between the clinical outcome measures and the symmetry indices (*S*_ML_, *S*_AP_, *S*_IS_) in the mediolateral (ML), anteroposterior (AP), and inferiosuperior (iS) center of mass (CoM) directions.

CoM direction		Clinical outcome measures					
		10MWT	2MWT	WISCI-II	SCI-FAI	Mini-BESTest	LEMS
Single-sine coefficients	*S*_ML_	0.90^▪^	**1**.**0**^▪^*	−0.45	0.62^⧫^	0.46	−0.30
	*S*_AP_	0.60^⧫^	0.30	−0.22	0.67^⧫^	−0.05	−0.80^▪^
	*S*_IS_	0.30	0.10	−0.45	0.31	−0.46	−0.90^▪^

Strong correlation values are indicated with a “▪”, and moderate correlations with a “⧫”. Bolded numbers with an asterisk (*) indicate the correlation is significantly different than zero (29). Significant level of *p* = 0.05 was used. 10MWT, 10Meter Walking Test; 2MWT, 2Minute Walking Test; WISCI-II, Walking Index for Spinal Cord Injury II; SCI-FAI, Spinal Cord Injury Functional Ambulation Index; Mini-BESTest, Mini Balance Evaluation System Test; LEMS, lower extremity motor score.

## 4 Discussion

### 4.1 Clinical outcome measures

No single routinely applied clinical gait assessment captured the totality of the impairments exhibited by our small sample of ambulatory individuals with iSCI. There were observable discrepancies among the clinical measures with respect to the severity of injury, which may stem from each assessment being sensitive to different aspects of gait. The LEMS solely evaluates muscle strength while the Mini-BESTest is intended to summarize aspects of balance, coordination, and sensation/proprioception. The SCI-FAI was designed to capture coordination, motor function, and gait quality, but is agnostic on other factors. Both the 10MWT and 2MWT reflect information related to the quality of motor function and aerobic capacity through the surrogates of gait speed and distance. The different presentations of each participant resulted in their scoring higher in one domain or another without being consistent with overall ambulatory function across all clinical measures.

The discrepancies may also stem from other underlying factors. SCI05 wears ankle foot orthoses (AFOs) since he lacks volitional dorsiflexion and has weak plantarflexion movement. This translated to a low Mini-BESTest since ankle control enhances stability, a low LEMS due to its inclusion of ankle strength, and a higher SCI-FAI score since the AFOs prevented toe drag. With each clinical measure masking such underlying issues, multiple tests are necessary to fully characterize iSCI gait, which still may not capture the multidimensional phenomenon in its entirety. Yet, CoM symmetry appears to correlate well with four of the clinical assessments, suggesting that it captures and integrates the salient information from each in one objective measurement.

### 4.2 Center of mass profiles

The CoM profile shape expectantly differentiated across ambulatory categories through visual inspection. SCI04, a limited community ambulator, had CoM patterns that were most similar to SCI03 and SCI02 who were identified as household ambulators. This was expected since SCI04's gait speed (0.41 m/s) is close to the upper definition for household, (<0.4 m/s), and lower border of limited community, (0.4–0.8 m/s) functional ambulatory categories (12). Additionally, visual inspection of the CoM profile shape (Figure 3) suggests that the order in which subjects resembled NT gait coincided with the polygon areas summarizing all clinical measures from Figure 2. This suggests that characteristics of the overall CoM may reflect gait impairments with a single metric and combine the information derived from multiple assessments. The exact nature of these morphological changes to the CoM with specific gait impairments, AT prescriptions, and progress through rehabilitation, remain to be determined and warrant closer investigation in larger and more detailed studies.

CoM appears sensitive to variation across levels of impairments/ambulatory categories due to compensatory strategies, like the unexpected shift toward the swing instead of stance limbs for SCI01 and SCI05. SCI01 may adopt this strategy to aid toe clearance, while SCI05 for balance due to the lack of ankle mobility. Supplementary Figure S2 shows results of twenty additional trials without crutches and with bilateral anterior shell AFOs by SCI05. Without crutches, he shifted expectedly towards the stance limb but with wider lateral sway for stability. In the frontal plane, SCI02 bent his right knee during stance, which manifested in an asymmetrical, flat CoM profile. SCI04 exhibited a shorter right stance compared to his left which may stem from weaker right hip and knee extensors and adopted a “limping” strategy to minimize reliance on his weaker limb ostensibly to protect against collapse. Neurotypical gait is relatively symmetrical (32), as shown in Figure 3 NT01 plot, so the asymmetries presented across the participants might be explained by asymmetrical motor function post-SCI.

The effects of compensatory strategies and gait deficits on CoM due to motor function, strength, and assistive devices provides a new component of information on gait that established clinical assessments otherwise lack. CoM might be able to inform clinicians about which muscles and/or gait phases to target to maximize the effects of rehabilitation and best approach NT gait patterns. For example, Figure 3 indicates that SCI02 and SCI04 had weak knee extensors that translated differently to the walking pattern. Therefore, a clinician may tailor interventions and/or exercise programs respectively to produce a rounder curve for SCI02 and a more symmetrical one for SCI04. CoM identified gait behaviors not readily observable by visual inspection, which could benefit clinical decision making. For instance, clinicians might use the information from these analyses to encourage SCI05 to shift over his stance foot in swing while utilizing forearm crutches, which would drive the CoM toward a more symmetrical NT pattern, and possibly reduce fall risk if the individual steps without crutches.

### 4.3 Center of mass parameterization

We focused analysis on the symmetry indices to 1. condense the three harmonics of the model into a single score, and 2. acknowledge and exploit the known association between gait symmetry and metabolic energy (20), which determines gait efficiency (33). This exploration suggests that both $S_{\mathrm{ML}}$ and $S_{\mathrm{AP}}$ correlate with gait speed. This was expected since CoM is known to vary with speed in both NT (13) and iSCI (17) gait. However, Bergamini et al. determined that gait speed significantly affects $S_{\mathrm{AP}}$ and $S_{\mathrm{IS}}$, but not $S_{\mathrm{ML}}$ (30). Our findings partly support this since we found a moderate correlation with $S_{\mathrm{AP}}$, but a low and strong correlation with $S_{\mathrm{IS}}$ and $S_{\mathrm{ML}}$, respectively. This may be because our participants walked overground vs. a treadmill (30) which exhibit different kinetics, kinematics, and electromyographic activities, impacting the CoM (34). Additionally, treadmills may impose imbalance, so participants implement a more cautious gait which might preferentially affect $S_{\mathrm{ML}}$. Another reason for this slight difference between studies may be due to our focus on iSCI vs. NT gait (30). Movements of the CoM in the AP and IS directions have evolved to prioritize the minimization of metabolic energy expenditure (19), which determines a preferred gait speed in NT individuals (35). However, individuals with iSCI have varying degrees of asymmetry in motor function and exhibit reduced muscle contractile speed (36). Such issues would impact the muscular effort required for persons with SCI to walk and may explain the significant effects gait speed had on $S_{\mathrm{IS}}$ in (30) and the weak correlations observed in our study. Dusane et al. demonstrated improved lateral control at higher walking speeds in individuals with SCI (17), which was confirmed by the $S_{\mathrm{ML}}$ observed in our study. Furthermore, based on the statistically significant findings in Dusane et al.*,* we may increase our confidence that the probability for type II error with our $S_{\mathrm{ML}}$ and 2MWT correlation is low.

The moderate correlation between the SCI-FAI score and $S_{\mathrm{ML}}/S_{\mathrm{AP}}$ we observed were also expected. The SCI-FAI includes scores for weight shifting and step width, which require ML control of the hip abductors/adductors (16). SCI02-SCI04 had asymmetrical strength for these muscles (Supplementary Figure S3). Furthermore, the SCI-FAI scores step rhythm, foot contact, and step length. These require AP control via the plantarflexors and dorsiflexors (16), and SCI01 and SCI03 had asymmetrical ankle strength. While step height implies control in the IS direction, the criterion for this parameter focuses on AP control with toe clearance through swing. With the SCI-FAI solely scoring ML and AP control, this may explain the weak correlation with $S_{\mathrm{IS}}$.

Unexpectedly, the results suggested that the LEMS was negatively correlated with $S_{\mathrm{ML}}$, $S_{\mathrm{AP}}$, and $S_{\mathrm{IS}}$. Negative correlations with $S_{\mathrm{AP}}$ and $S_{\mathrm{IS}}$ were strong, although weak with $S_{\mathrm{ML}}$. In theory, we would expect a strong, positive correlation with muscle strength because its association with joint moments (6), gait speed (37), and mechanical energy expenditure (38), which all contribute to efficient movement of the CoM (17, 20, 39). Standard methodology for applying the LEMS itself may mask any strength differences between sides which may affect the CoM indices (24), such as its lack of consideration of hip ab/adduction (16). Future investigations should include all lower extremity muscle movements and either computing a strength symmetry index such as the symmetry ratio (40) or report separate right and left scores.

Unsurprisingly, the WISCI-II had a weak correlation with the CoM Indices because it primarily scored the type of AT. Overcompensation and reduction in the required strength to achieve stepping due to AT may explain the negative correlation (6). For instance, SCI05 had a low WISCI-II, but high CoM Indices. His bilateral AFOs helped stabilize his ankles equally and possibly aided his gait symmetry. A walker or parallel bars (i.e*.,* lower WISCI-II scores) may have a similar effect on CoM symmetry due to their bilateral design.

Surprisingly, the Mini-BESTest and CoM Indices were weakly correlated, despite CoM being associated with dynamic stability in NT (41, 42) and pathological (17, 43) gait. $S_{\mathrm{ML}}$ was the closest to achieving a moderate correlation, which is expected since ML CoM prioritizes balance (17). The reliance on ADs may have contributed to the weak correlation since they affect the base of the support (44) which interacts with CoM to define static and dynamic stability (42), thereby minimizing possible asymmetries. The weak correlations require further investigation, and we suggest including less impaired participants who do not require ADs to further understand this phenomenon. With a larger sample size that addresses the absence of ADs, we would expect a shift towards a stronger correlation with the $S_{\mathrm{ML}}$ since CoM correlates with balance (17). We can partially accept our hypothesis since we observed moderate to strong correlations between the CoM symmetry indices and only the 10MWT, 2MWT, and LEMS.

### 4.4 Future directions and clinical applications

Further study could investigate whether the model parameters for particular gait impairments cluster into discernable groups in feature space to aid in diagnosis or monitoring progress via the movement of subsequent clusters toward a cluster indicative of NT walking. Similarly, the phase angles of the functions in the CoM model were not part of our analysis and reflect relative timing which could indicate whether a feature of pathological gait is leading or lagging NT and needs to be delayed or advanced relative to other gait events. Thus, exploring the information content of the CoM profile captured by the parameters of the CoM model should be a priority for future work so it could best be incorporated into clinical decision making.

Although the results of this study are preliminary, they indicate the potential for utilizing CoM position as an initial screening tool, as it could add information to gait evaluations not readily observed by a clinician and identify targeted muscle groups for preparatory exercises, or other priorities for rehabilitation and gait training. However, much work remains before the CoM profile approach could be translated to the clinic. CoM profiles need to be estimated in real time from a convenient and unobtrusive set of wireless wearable inertial measurement units (IMUs), or from a single sensor serving as a surrogate (45, 46). Tablet or laptop based clinical software could be developed to receive wireless data transmitted from the sensors and generate ensemble CoM profiles and compute the symmetry indices, or other parameters of interest. This would allow clinicians to visually discern and quantify gait deficits and guide intervention selection. Since wearable wireless IMUs and processing algorithms are rapidly becoming ubiquitous in consumer electronic and health monitoring devices such as Fitbits, smart watches, cellphones and the like, such developments are feasible with current technologies.

### 4.5 Limitations of current study

One limitation of this exploratory study is the small sample size. While our results suggested moderate to strong correlations, larger scale trials that better represent the population of ambulatory individuals with iSCI are needed to generalize and fully determine the utility of the CoM approach. Another limitation is the variable age range with our participant pool. CoM acceleration and work have been shown to differ between age groups and may stem from muscle activation changes (31). Since musculoskeletal control is compromised after SCI, the exact contribution age has on CoM in the SCI population remains unclear. Another limitation is only having one female participant in this study. Knee joint angles and pelvic obliquity contribute to changes in CoM kinematics (47) and significantly differ between NT males and females (48). Potential confounds such as gender and other factors need to be rigorously examined to fully validate the approach and account for the high degree of intersubject variability in the iSCI population. Although SCI01 was the sole female in our cohort, her data may have been influenced as much by her medical history as by gender or other considerations. Her previous bilateral hinged knee replacements (49) could have contributed to her characteristically atypical CoM profile and symmetry indices. Another limitation is our utilization of the first three harmonics of the CoM model for computational efficiency and consistency across subjects. Further research is needed to identify the minimum number of harmonics to include in subject-specific models that capture all salient features of the CoM as a function of clinical characteristics such as injury level and other impairments to baseline walking abilities.

## 5 Conclusion

This exploratory study characterized CoM position in a small number of ambulatory individuals with iSCI, and suggests the feasibility of such a new, comprehensive method to objectively and quantitatively assess walking performance that incorporates valuable features of several clinically accepted observational gait evaluations in a single global variable. Well-established clinical measures of gait performance are sensitive to various aspects of strength, coordination, balance, aerobic capacity, and motor function, but appear to not singularly capture the multidimensionality of iSCI gait in its entirety. The CoM profile may integrate many of the variables evaluated by clinical assessments to support global, quantitative, and objective insight into walking performance. Symmetry indices derived from a mathematical representation of CoM profile in all three directions appear to be correlated with many clinically accepted subjective assessments of gait quality, speed, and muscle strength. For example, $S_{\mathrm{ML}}$ may be correlated with gait speed and quality, while $S_{\mathrm{AP}}$ correlated with gait speed, quality, and muscle strength, and $S_{\mathrm{IS}}$ correlated with muscle strength alone. These unique combinations emphasize the importance of all three CoM components in the understanding of gait quality and motor control, and suggest that CoM position may capture similar information as the four separately applied clinical assessments. The 3D CoM profile describes whole-body movement and appears to reflect distinct changes in gait performance with increasing impairment and injury severity due to iSCI, with its associated compensatory strategies and use of ADs. The unique features of parameterized 3D CoM profiles and their correlations with clinical assessments may suggest clinical relevance and the potential to complement current methods of gait evaluation in the clinical setting. To bring this potential to fruition and establish generalizability, a larger scale trial representing a broader range of ambulatory individuals with iSCI is required, and efficient methods to implement CoM analysis in the clinic without specialized equipment or computational resources still need to be developed.

## Data Availability

The raw data supporting the conclusions of this article will be made available by the authors, without undue reservation.

## References

[1] Ferber GA Anderson KD. Recovery insights following spinal cord injury. Phys Med Rehabil Clin N Am. (2025) 36(1):139–54. 10.1016/j.pmr.2024.08.002 39567032

[2] Patrick M Ditunno P Ditunno JF Marino RJ Scivoletto G Lam T Consumer preference in ranking walking function utilizing the walking index for Spinal Cord Injury II. Spinal Cord. (2011) 49(12):1164–72. 10.1038/sc.2011.77 21788954

[3] Lo C Tran Y Anderson K Craig A Middleton J. Functional priorities in persons with spinal cord injury: using discrete choice experiments to determine preferences. J Neurotrauma. (2016) 33:1958–68. 10.1089/neu.2016.4423 27080545

[4] Morris JN Hardman AE. Walking to health. Sports Med. (1997) 23:306–32. 10.2165/00007256-199723050-00004 9181668

[5] Perry J. Gait Analysis: Normal and Pathological Function. 2nd ed. Thorofare, NJ: Slack Incorporated (1992). p. 2010.

[6] Barbeau H Nadeau S Garneau C. Physical determinants, emerging concepts, and training approaches in gait of individuals with Spinal Cord Injury. J Neurotrauma. (2006) 23:571–85. 10.1089/neu.2006.23.571 16629638

[7] Werner C Gönel M Lerch I Curt A Demkó L. Data-driven characterization of walking after a spinal cord injury using inertial sensors. J Neuroeng Rehabil. (2023) 20:55. 10.1186/s12984-023-01178-9 37120519 PMC10149024

[8] Lam T Noonan VK Eng JJ. A systematic review of functional ambulation outcome measures in spinal cord injury. Spinal Cord. (2008) 46:246–54. 10.1038/sj.sc.3102134 17923844 PMC3095631

[9] Bolliger M Blight AR Field-Fote EC Musselman K Rossignol S Barthélemy D Lower extremity outcome measures: considerations for clinical trials in Spinal Cord Injury. Spinal Cord. (2018) 56:628–42. 10.1038/s41393-018-0097-8 29700477 PMC6131138

[10] Jørgensen V Opheim A Halvarsson A Franzén E Roaldsen KS. Comparison of the Berg balance scale and the mini-bestest for assessing balance in ambulatory people with Spinal Cord Injury: validation study. Phys Ther. (2017) 97:677–87. 10.1093/ptj/pzx030 28371940

[11] Hoffer MM Feiwell E Perry R Perry J Bonnett C. Functional ambulation in patients with Myelomeningocele. J Bone Joint Surg. (1973) 55:137–48. 10.2106/00004623-197355010-00014 4570891

[12] Schmid A Duncan PW Studenski S Lai SM Richards L Perera S Improvements in speed-based gait classifications are meaningful. Stroke. (2007) 38:2096–100. 10.1161/STROKEAHA.106.475921 17510461

[13] Tesio L Rota V. The motion of body center of mass during walking: a review oriented to clinical applications. Front Neurol. (2019) 10:999. 10.3389/fneur.2019.00999 31616361 PMC6763727

[14] do Carmo A Kleiner A Barros R. Alteration in the center of mass trajectory of patients after stroke. Top Stroke Rehabil. (2015) 22:349–56. 10.1179/1074935714Z.0000000037 25906834

[15] Merello M Fantacone N Balej J. Kinematic study of whole body center of mass position during gait in Parkinson’s disease patients with and without festination. Mov Disord. (2010) 25:747–54. 10.1002/mds.22958 20222128

[16] Winter D. Biomechanical movement synergies. In: Winter D, editors. Biomechanics and Motor Control of Human Movement. 4th ed. Hoboken, NJ: John Wiley & Sons, Inc (2009). p. 281–95.

[17] Dusane S Shafer A Ochs WL Cornwell T Henderson H Kim K-YA Control of center of mass motion during walking correlates with gait and balance in people with incomplete spinal cord injury. Front Neurol. (2023) 14:1146094. 10.3389/fneur.2023.1146094 37325225 PMC10262050

[18] Lemay J-F Duclos C Nadeau S Gagnon D Desrosiers É. Postural and dynamic balance while walking in adults with incomplete spinal cord injury. J Electromyogr Kinesiol. (2014) 24:739–46. 10.1016/j.jelekin.2014.04.013 24909105

[19] Wurdeman SR Raffalt PC Stergiou N. Reduced vertical displacement of the center of mass is not accompanied by reduced oxygen uptake during walking. Sci Rep. (2017) 7:17182. 10.1038/s41598-017-17532-6 29215063 PMC5719393

[20] Minetti AE Cisotti C Mian OS. The mathematical description of the body centre of mass 3D path in human and animal locomotion. J Biomech. (2011) 44:1471–77. 10.1016/j.jbiomech.2011.03.014 21463861

[21] Kumprou M Amatachaya P Sooknuan T Thaweewannakij T Mato L Amatachaya S. Do ambulatory patients with Spinal Cord Injury Walk symmetrically? Spinal Cord. (2017) 55:204–7. 10.1038/sc.2016.149 27824056

[22] Shirley Ryan Abilitylab. International Standards for Neurological Classification of Spinal Cord Injury (Asia Impairment Scale). Chicago, IL: Shirley Ryan AbilityLab (2013). Available online at: https://www.sralab.org/rehabilitation-measures/international-standards-neurological-classification-spinal-cord-injury-asia-impairment-scale.

[23] Chaffin DB Anderson GBJ Martin BJ. Occupational Biomechanics. 4th ed. Hoboken, NJ: John Wiley & Sons, Inc (2006). p. 37–53.

[24] Kendall FP McCreary EK Provance PG Rodgers MM Romani WA. Muscles, Testing and Function with Posture and Pain. 5th ed. Baltimore, MD: Lippincott Williams & Wilkins (2005).

[25] Burns S Biering-Sorensen F Donovan W Graves D Jha A Johansen M International standards for neurological classification of Spinal Cord Injury, revised 2011. Top Spinal Cord Inj Rehabil. (2012) 18:85–99. 10.1310/sci1801-85 23460761 PMC3584745

[26] Vicon. Plug-in Gait Reference Guide. Oxford: Vicon Motion Systems (2023). Available online at: https://help.vicon.com/space/Nexus216/11607059/Plug-in+Gait+Reference+Guide

[27] Labrozzi GC Warner H Makowski NS Audu ML Triolo RJ. Center of mass estimation for impaired gait assessment using inertial measurement units. IEEE Trans Neural Syst Rehabil Eng. (2024) 32:12–22. 10.1109/TNSRE.2023.3341436 38090847 PMC10849874

[28] Schober P Boer C Schwarte LA. Correlation coefficients: appropriate use and interpretation. Anesth Analg. (2018) 126:1763–68. 10.1213/ane.0000000000002864 29481436

[29] Corr. Linear or Rank Correlation. Natick, MA: MATLAB. (2024). Available online at: https://www.mathworks.com/help/stats/corr.html (Accessed December 1, 2024).

[30] Bergamini E Cereatti A Pavei G. Walking symmetry is speed and index dependent. Sci Rep. (2024) 14:19548. 10.1038/s41598-024-69461-w 39174605 PMC11341956

[31] Hernández A Silder A Heiderscheit BC Thelen DG. Effect of age on center of mass motion during human walking. Gait Posture. (2009) 30:217–22. 10.1016/j.gaitpost.2009.05.006 19502061 PMC3199953

[32] Crosby LD Chen JL Grahn JA Patterson KK. Perceptions of an over-ground induced temporal gait asymmetry by healthy young adults. Hum Mov Sci. (2021) 78:102806. 10.1016/j.humov.2021.102806 34020406

[33] Winter D. Mechanical Work, Energy, and Power. in Biomechanics and Motor Control of Human Movement. 4th ed. Hoboken, NJ: John Wiley & Sons, Inc (2009). p. 149–54.

[34] Semaan MB Wallard L Ruiz V Gillet C Leteneur S Simoneau-Buessinger E. Is treadmill walking biomechanically comparable to overground walking? A systematic review. Gait Posture. (2022) 92:249–57. 10.1016/j.gaitpost.2021.11.009 34890914

[35] Sasaki K Neptune RR. Muscle mechanical work and elastic energy utilization during walking and running near the preferred gait transition speed. Gait Posture. (2006) 23:383–90. 10.1016/j.gaitpost.2005.05.002 16029949

[36] Thomas CK Bakels R Klein CS Zijdewind I. Human spinal cord injury: motor unit properties and behaviour. Acta Physiol. (2014) 210:5–19. 10.1111/apha.12153 23901835

[37] Abdul Jabbar K Seah W-T Lau LK Pang BW-J Ng DH-M Tan QL-L Fast gait spatiotemporal parameters in adults and association with muscle strength—the Yishun study. Gait Posture. (2021) 85:217–23. 10.1016/j.gaitpost.2021.01.001 33610825

[38] McGibbon CA Puniello MS Krebs DE. Mechanical energy transfer during gait in relation to strength impairment and pathology in elderly women. Clin Biomech. (2001) 16:324–33. 10.1016/s0268-0033(01)00004-3 11358620

[39] Kwon S Oh Y. Estimation of the center of mass of humanoid robot. 2007 International Conference on Control, Automation and Systems; Oct 2007; Seol, Korea (South). p. 2705–9. 10.1109/ICCAS.2007.4406826

[40] Cabral S. Gait symmetry measures and their relevance to gait retraining. In: Muller B, Wolf SI, editors. Handbook of Human Motion. Cham: Springer International Publishing AG (2018). p. 429–47. 10.1007/978-3-319-14418-4_201

[41] Jurcevic T Muftic O. Trajectory of the human body mass centre during walking at different speed. International Design Conference—Design 2002; 2022 May 14–17; Dubrovnik, Croatia.

[42] Lugade V Lin V Chou L-S. Center of mass and base of support interaction during gait. Gait Posture. (2011) 33:406–11. 10.1016/j.gaitpost.2010.12.013 21211977

[43] Vistamehr A Kautz SA Bowden MG Neptune RR. Correlations between measures of dynamic balance in individuals with post-stroke hemiparesis. J Biomech. (2016) 49:396–400. 10.1016/j.jbiomech.2015.12.047 26795124 PMC4761510

[44] Faruqui SR Jaeblon T. Ambulatory assistive devices in orthopaedics: uses and modifications. Am Acad Orthop Surg. (2010) 18:41–50. 10.5435/00124635-201001000-00006 20044491

[45] Cho Y-S Jang S-H Cho J-S Kim M-J Lee HD Lee SY Evaluation of validity and reliability of inertial measurement unit-based gait analysis systems. Ann Rehabil Med. (2018) 42:872–83. 10.5535/arm.2018.42.6.872 30613081 PMC6325313

[46] Cardarelli S Mengarelli A Tigrini A Strazza A Di Nardo F Fioretti S Single IMU displacement and orientation estimation of human center of mass: a magnetometer-free approach. IEEE Trans Instrum Meas. (2020) 69:5629–39. 10.1109/tim.2019.2962295

[47] Hayot C Sakka S Lacouture P. Contribution of the six major gait determinants on the vertical center of mass trajectory and the vertical ground reaction force. Hum Mov Sci. (2013) 32:279–89. 10.1016/j.humov.2012.10.003 23725827

[48] Cho SH Park JM Kwon OY. Gender differences in three dimensional gait analysis data from 98 healthy Korean adults. Clin Biomech. (2004) 19:145–52. 10.1016/j.clinbiomech.2003.10.003 14967577

[49] Makowski NS Lombardo LM Foglyano KM Kobetic R Pinault G Selkirk SM Walking after incomplete spinal cord injury with an implanted neuromuscular electrical stimulation system and a hinged knee replacement: a single-subject study. Spinal Cord Ser. Cases. (2020) 6:86. 10.1038/s41394-020-00336-8 32934207 PMC7493919

[50] Hnat SK Audu ML Triolo RJ Quinn RD. Estimating center of mass kinematics during perturbed human standing using accelerometers. J Appl Biomech. (2021) 37:415–24. 10.1123/jab.2020-0222 34453018

[51] Gupta A Stead TS Ganti L. Determining a meaningful R-squared value in clinical medicine. Acad Med Surg. (2024). 10.62186/001c.125154

