# Supplementary material for: Exploration of the center of mass kinematics correlation with established gait measures in post-spinal cord injury
Source: Front Rehabil Sci. 2026 May 11;7:1745887. doi: 10.3389/fresc.2026.1745887 (PMC13199266; doi:10.3389/fresc.2026.1745887)
Supplement: Supplementary file 1 [file Datasheet1.docx]

Supplementary Material

# Supplementary Methods: Computation of Symmetry Indices

To compute the symmetry indices, we utilized the mathematical model, which defines CoM position as:

$CoM_{x,y,z}\left( t \right)=A_{0x,y,z}+\sum_{i=1}^{N} c_{i}^{x,y,z}\sin(2\pi fit+\phi_{iR}^{x,y,z})$ (1)

Where $CoM_{x,y,z}(t)$ denotes the CoM components in the x (ML), y (AP), and z (IS) directions at any instant, $c_{i}^{x,y,z}$ are the single-sine coefficients for each direction, $A_{0x,y,z}$are terms to align the start of the signal at position $\left( 0,0,0 \right)$, $f$ is the stride frequency, $t$ is the absolute chronological time, $i$ is the harmonic (or term) number, $N$ is the total number of harmonics ($N\in\left[ 1,6 \right]$), and $\phi_{iR}^{x,y,z}$ are the phase angles relative to the phase angle in the ML direction as defined in (20).

Inspired by the methods reported in Minetti *et al.,* we identified the parameters of the model for each subject from their experimental data with a non-linear gradient-based optimizer (Levenberg-Marquardt algorithm) from the *MATLAB Optimization Toolbox* (The MathWorks, Inc., Natick, MA) specifically intended for parameter estimation. The objective function ($E\left( t \right)_{total}$) was set to minimize the sum of the squared errors between the experimental (original) and predicted (model) CoM positions:

$E\left( t \right)_{x,y,z}=\left( \sum_{t=1}^{n} {(CoM_{Org_{x,y,z}}\left( t \right)-{CoM}_{mod_{x,y,z}}\left( t \right))}^{2} \right)$ (2)

$E\left( t \right)_{total}=E\left( t \right)_{x}+ E\left( t \right)_{y}+ E\left( t \right)_{z}$ (3)

Where $CoM_{Org_{x,y,z}}\left( t \right)$ denotes the experimental CoM signal across a given stride time $\left( t \right)$ in the ML (x), AP (y), and IS (z) directions, $CoM_{mod_{x,y,z}}\left( t \right)$ is the reconstructed (model) CoM, and $n$ is the total number of discrete time points.

K-fold cross-validations were performed to determine the appropriate number of harmonics to avoid under/overfitting the data. For each participant, strides were randomized and further shuffled into 10 folds with 70% used for training and 30% for validation per fold (51). R^2^ values between the mean experimental and model-predicted CoM positions were computed in each direction for increasing number of harmonics. A minimum R^2^ value of 0.7 was considered acceptable (52) and overfitting indicated by a decrease in the validation R^2^ across harmonics. Once the optimal number of harmonics was determined, the truncated model was refit to data from all strides, and symmetry indices derived from the resulting single-sine coefficients.

$S_{ML}=\frac{c_{1}^{x}+c_{3}^{x}+c_{5}^{x}}{\sum_{i=1}^{6} c_{i}^{x}}$ , $S_{AP}=\frac{c_{2}^{y}+c_{4}^{y}+c_{6}^{y}}{\sum_{i=1}^{6} c_{i}^{y}}$ , $S_{IS}=\frac{c_{2}^{z}+c_{4}^{z}+c_{6}^{z}}{\sum_{i=1}^{6} c_{i}^{z}}$ (4)

where perfect symmetry is equivalent to an index value of unity (20).

# Supplementary Figures and Tables

## Supplementary Figures

**(A)**


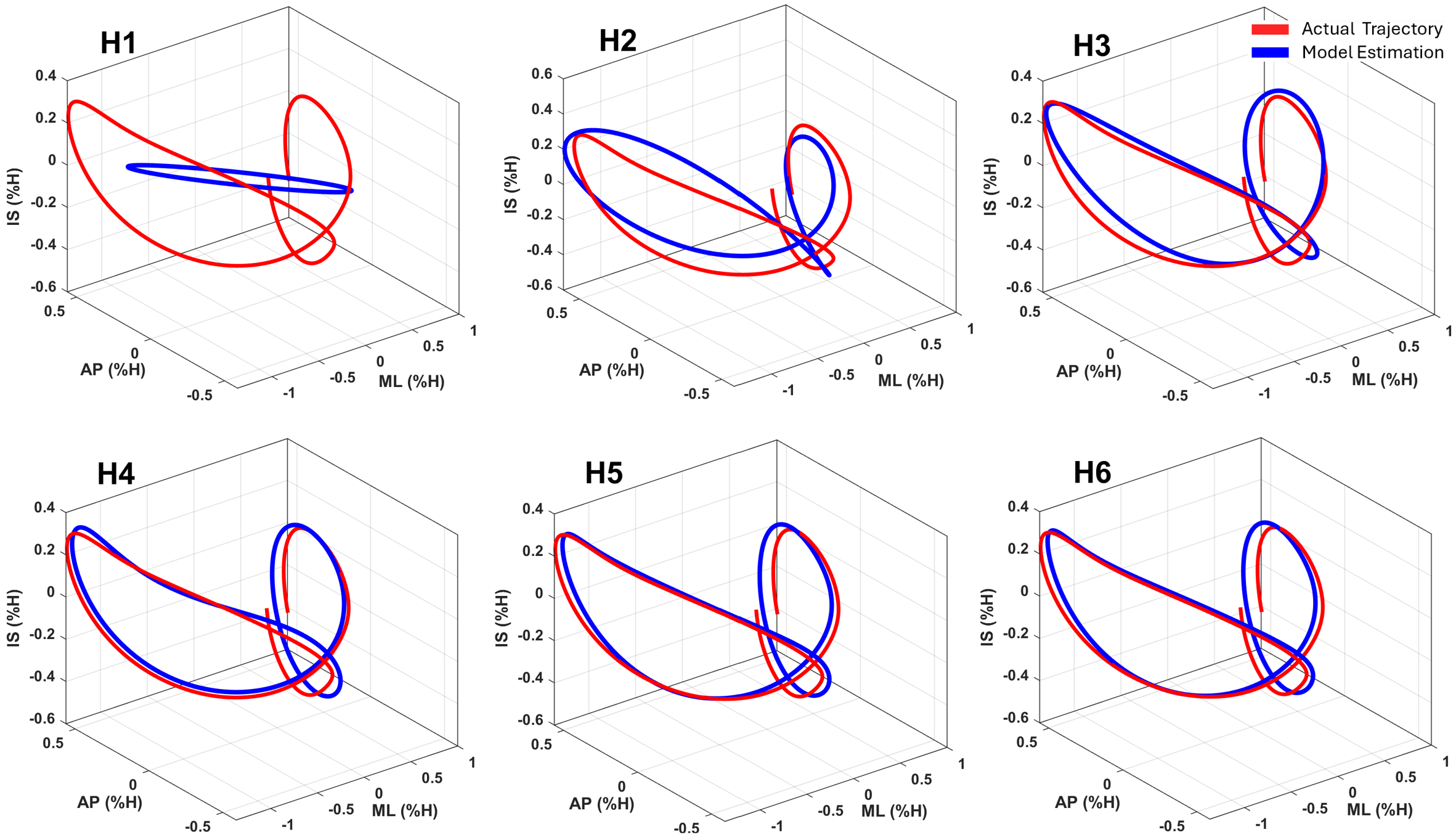


**(B)**


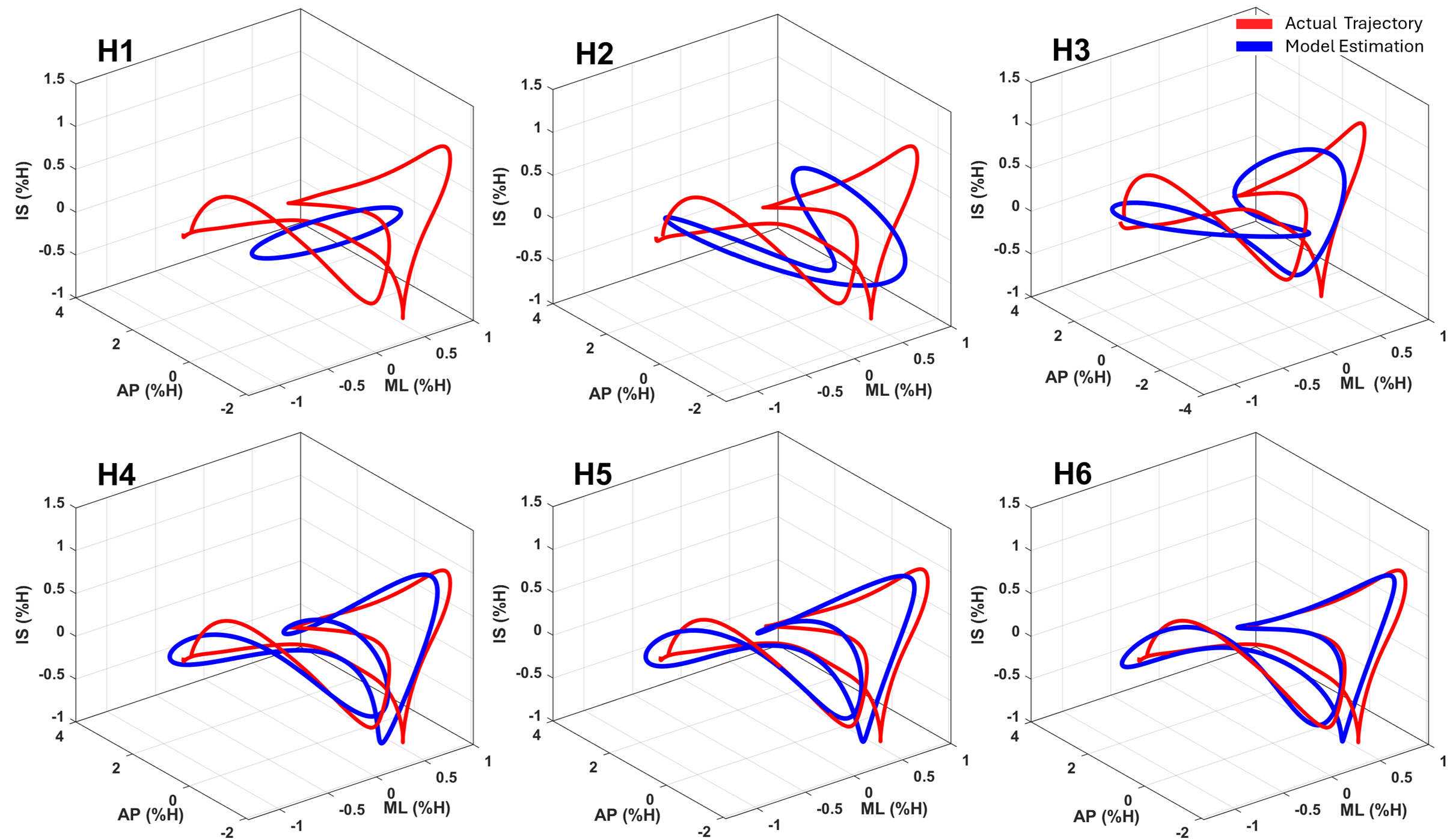


**SUPPLEMENTARY FIGURE 1A-B**

The 3D representations of the model’s estimation of CoM trajectory for SCI03 (A, top) and SCI01 (B, bottom) across the 6 harmonics (H1-H6). The CoM signals are plotted against each other over the average stride. The red line is the actual CoM trajectory for one validation set from the 10-fold sets. The blue line is the Model’s estimation of the CoM signal for the given set.


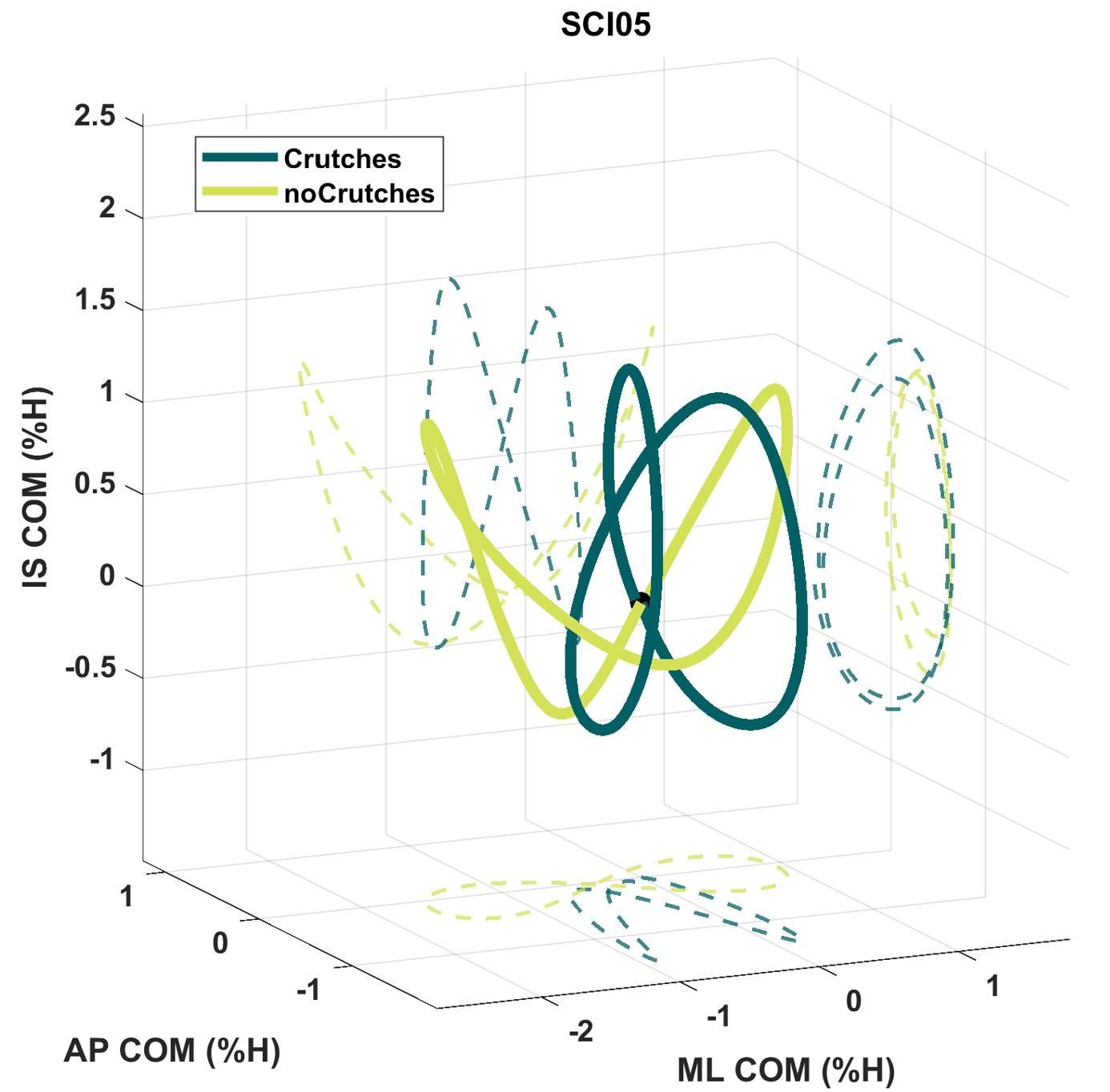


**SUPPLEMENTARY FIGURE 2**

Assisted Devices Impact on CoM. The 3D representations of the CoM trajectory for SCI05 with and without crutches over the average stride (solid line). The dashed line is the average CoM projected onto each plane. The black dot represents the starting point (right heelstrike). Each trajectory is normalized to the subject’s height and depicted as a percent.


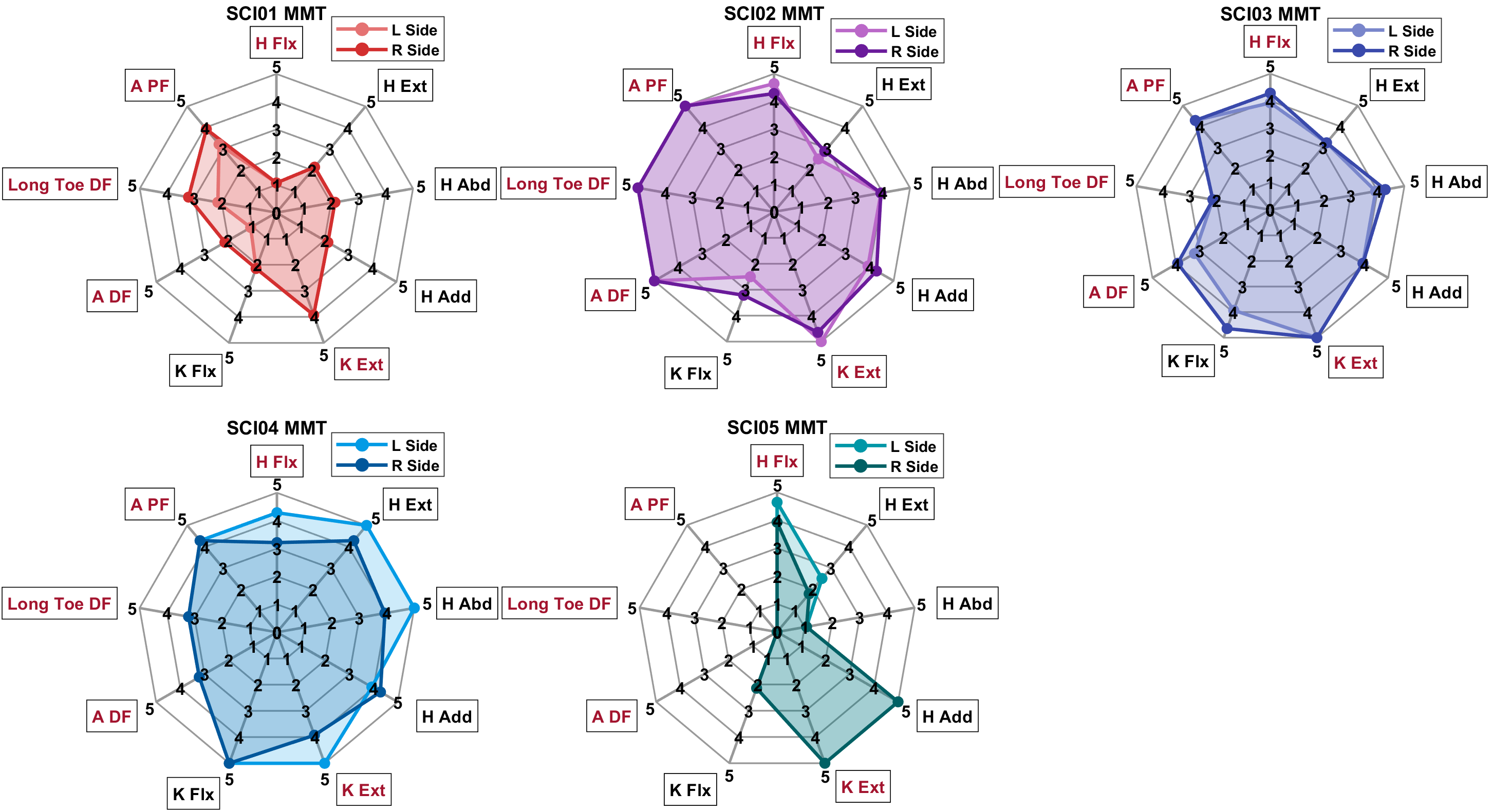


**SUPPLEMENTARY FIGURE 3:**

Spiderplot of Muscle Manual Testing. The MMT scores for SCI01-05 for nine muscle movements. We used a 14-point scale that ranged from 0-5 which included pluses and minuses for higher precision between the right (dark color) and left (light color) side. The muscle movements highlighted in red indicate the muscle groups used to calculate the LEMS score. H Flx = Hip Flexors; H Ext = Hip Extensors; H Abd = Hip Abduction; H Add = Hip Adduction; K Ext = Knee Extensors; K Flx = Knee Flexors; A DF = Ankle Dorsiflexors; Long Toe DF = Long Toe Dorsiflexors; A PF = Ankle Plantarflexors.

## Supplementary Tables

SUPPLEMENTARY TABLE 1: The average R^2^ value between the actual and estimated CoM trajectory for the 3^rd^ Harmonic.

| **Subject** | **Type of Assisted Device** | **R^2^ ML** | **R^2^ AP** | **R^2^ IS** |
| --- | --- | --- | --- | --- |
| SCI 01 | Forearm Crutches | 0.89 | 0.87 | 0.45 |
| SCI 02 | Forearm Crutches | 0.96 | 0.93 | 0.94 |
| SCI 03 | Forearm Crutches | 0.88 | 0.94 | 0.94 |
| SCI 04 | Cane – Right Side | 0.93 | 0.94 | 0.93 |
| SCI 05 | Forearm Crutches, Posterior Shell AFOs | 0.92 | 0.99 | 0.99 |
| SCI 05 | Anterior Shell AFOs | 0.83 | 0.96 | 0.99 |

SCI05 completed an additional 20 trials without crutches and with different AFOs.
